# Clinical Application of Volumetric Absorptive Microsampling for Therapeutic Drug Monitoring of Oral Targeted Anticancer Drugs

**DOI:** 10.1097/FTD.0000000000001315

**Published:** 2025-02-25

**Authors:** Marinda Meertens, Nikki Kerssemakers, Niels de Vries, Hilde Rosing, Neeltje Steeghs, Jos H. Beijnen, Alwin D. R. Huitema

**Affiliations:** * Department of Pharmacy and Pharmacology, The Netherlands Cancer Institute–Antoni van Leeuwenhoek Hospital, Amsterdam, the Netherlands;; † Department of Medical Oncology, The Netherlands Cancer Institute–Antoni van Leeuwenhoek Hospital, Amsterdam, the Netherlands;; ‡ Department of Medical Oncology, Utrecht University Medical Centre, Utrecht, the Netherlands;; § Division of Pharmacoepidemiology and Clinical Pharmacology, Utrecht Institute for Pharmaceutical Sciences, Utrecht University, Utrecht, the Netherlands;; ¶ Department of Clinical Pharmacy, University Medical Center Utrecht, Utrecht University, Utrecht, the Netherlands; and; ‖ Department of Pharmacology, Princess Máxima Center for Pediatric Oncology, Utrecht, the Netherlands.

**Keywords:** volumetric absorptive microsampling, therapeutic drug monitoring, LC-MS/MS, oral targeted anticancer drugs

## Abstract

**Background:**

Therapeutic drug monitoring optimizes oral anticancer drug treatment by measuring plasma levels. Volumetric absorptive microsampling (VAMS) allows home sampling with a minimal blood sample. However, methods for converting whole blood into plasma are required to interpret these results. This study aimed to establish conversion methods for abiraterone, alectinib, cabozantinib, imatinib, olaparib, sunitinib, and their metabolites, while assessing the differences between venous and capillary blood. The feasibility of home sampling was also evaluated.

**Methods:**

Plasma and VAMS samples, both from venipuncture-collected whole blood tubes and from a finger prick, were collected from each patient. The VAMS samples were deemed comparable if their concentrations were within ±20% of each other for ≥2/3rd of the patients. The Passing–Bablok regression and conversion factor methods were tested for the plasma and VAMS finger prick samples. The estimated plasma concentrations using both methods were required to be within ±20% of the measured plasma concentrations for ≥2/3rd of the pairs.

**Results:**

Overall, 153 patients were enrolled in this study. Conversion methods were applied to the VAMS samples, and the acceptance criteria were met for alectinib-M4, cabozantinib, imatinib, N-desmethyl imatinib, olaparib, sunitinib, and N-desethyl sunitinib but not for abiraterone, D4A, or alectinib. The capillary and venous VAMS concentrations were similar, except for that of D4A. Patients were positive toward home sampling.

**Conclusions:**

The established VAMS conversion methods for 7 out of 10 oral targeted anticancer drugs or metabolites met the acceptance criteria. Future studies need to validate the conversion methods with an independent cohort and integrate home sampling via VAMS to provide patients with an alternative to venipuncture at the outpatient clinic.

## Abstract 2

Supplemental Digital Content is Available in the Text.

## INTRODUCTION

Oral targeted anticancer drugs are important components in the treatment of various types of cancer. The combination of fixed-dose administration and complex pharmacokinetics results in high interindividual variability in plasma concentrations, leading to underdosing (>30% of patients) or overdosing (>15% of patients).^1^ This results in reduced antitumor activity or greater toxicity. Owing to this interpatient variability, therapeutic drug monitoring (TDM) is a feasible strategy for personalizing and improving treatment outcomes.^2^ TDM is recommended as a standard practice for some drugs, such as abiraterone, imatinib, and sunitinib. For other drugs, such as alectinib, cabozantinib, and olaparib, TDM-guided dosing is classified as promising.^3^ In addition to the recommendation to perform TDM as standard practice, it could also be useful for special indications, such as expected interactions or malabsorption.

TDM of oral targeted anticancer agents is performed by collecting blood via venipuncture and measuring the plasma concentration. This collection method has some disadvantages, including hospital visits, random sampling times resulting in the extrapolation of trough levels, fear of needles, and pain. Microsampling can overcome these limitations by collecting a small amount of capillary blood from the finger and is, therefore, less invasive and less painful. Furthermore, home sampling of patients is possible.^4^ Volumetric absorptive microsampling (VAMS) absorbs a fixed volume and thus, compared to other microsampling techniques such as dried blood spots, mitigates the potential impact of hematocrit on measurements.^4,5^

Certain elements must be addressed before the VAMS can be applied in clinical practice. VAMS collects capillary whole blood, whereas drugs of interest and their therapeutic thresholds are usually measured and established in plasma. Therefore, conversion methods must be developed to interpret the VAMS results. Blood-to-plasma ratios have already been determined in a clinical validation setting using VAMS or dried blood spot (DBS) for some oral targeted anticancer drugs, including cabozantinib, imatinib, and olaparib, but have to be determined for abiraterone, alectinib, sunitinib, and their metabolites.^6–10^ In the analytical validation of the VAMS quantification method for these 6 drugs with metabolites, ethylenediaminetetraacetic acid (EDTA) whole blood was used for calibrator and quality control preparation, assuming similarity to capillary blood upon reaching distribution equilibrium, although this requires verification.^11–13^ Furthermore, the feasibility of collecting VAMS samples by the patients themselves showed promising results, but further testing is needed to explore the potential of home sampling in this specific population.^7,14–16^

This study aimed to validate the clinical application of VAMS for the TDM of 6 oral targeted anticancer drugs, including their active metabolites abiraterone and D4A, alectinib and alectinib-M4, cabozantinib, imatinib, N-desmethyl imatinib, olaparib, sunitinib, and N-desethyl sunitinib. Therefore, conversion methods were determined for each analyte, and the difference between venously drawn EDTA blood and capillary-drawn blood was examined. Furthermore, the feasibility of home sampling was evaluated.

## MATERIALS AND METHODS

### Study Population

This study was conducted from February 2023 to August 2023 at the Netherlands Cancer Institute—Antoni van Leeuwenhoek Hospital. Patients treated with abiraterone, alectinib, cabozantinib, imatinib, olaparib, or sunitinib were considered eligible for the study. Patients were not eligible if they were undergoing concurrent chemotherapy, were hospitalized, or if blood sampling did not occur at the outpatient clinic. The treating physician assessed whether the patients were eligible to participate. The Medical Ethical Committee (Amsterdam, The Netherlands) approved the collection of data and the extra finger prick (reference number NL26128.048.08). Written informed consent was obtained from all the patients. The study adhered to the principles of the Declaration of Helsinki.

### Study Design

The first objective of this study was to examine the differences between VAMS from venously drawn EDTA whole blood tubes and VAMS capillary blood drawn from the finger. The second objective was to determine the methods for converting VAMS whole blood to plasma. The third objective was to evaluate the feasibility in a home sampling setting.

To address these objectives, the following study design was developed based on the International Association of Therapeutic Drug Monitoring and Clinical Toxicology guidelines for the development and validation of DBS-based methods for TDM.^12^ Blood samples were collected via venipuncture for routine TDM. Immediately after venipuncture, capillary blood was collected in duplicate using the VAMS device [Mitra 10 µL from Neoteryx (Torrance, CA)]. Becton Dickinson Microtainer contact-activated lancet of 2.0 mm was used for the finger prick. The sampling protocol was the fingers were disinfected using an alcohol wipe before the finger prick procedure. The first drop of blood was removed using a gauze pad before the 2 VAMS devices were filled with capillary blood. Two more VAMS samples were collected by dipping in EDTA tubes containing venous blood. This tube was centrifuged within an hour of collection, after which the plasma was transferred to a 3-mL tube. Three different samples were collected per patient in the hospital: K_2_EDTA plasma obtained by venipuncture, VAMS collected from the K_2_EDTA tube (in duplicate), and VAMS via a finger prick (in duplicate). VAMS samples collected after finger prick are referred to as “VAMS finger prick,” whereas those prepared from venous blood in tubes are referred to as “VAMS tube.”

#### Storage

Plasma samples were protected from light using amber-colored tubes. The VAMS samples were packaged in a plastic clamshell in an aluminum specimen bag protected from light, including a desiccant, and dried for at least 12 hours. After drying, the absorptive VAMS tip was collected in a 2-mL Eppendorf cup. Both the plasma and VAMS samples were stored in a freezer at −20°C until analysis.

### Bioanalysis

Samples were analyzed using validated Liquid Chromatography Tandem Mass Spectrometry methods.^13,17–20^ For the VAMS samples, compounds were extracted from the VAMS tip with methanol while shaking with grinding balls (3.96 mm outside diameter), evaporated with nitrogen, and reconstituted in acetonitrile: 100 mM ammonium hydroxide in water (1:1, v/v). Plasma samples were prepared by protein precipitation in acetonitrile. Both extracts were injected into separate but comparable Liquid Chromatography Tandem Mass Spectrometry systems, and separation was achieved on a reversed-phase column with detection using a triple quadrupole mass spectrometer operating in the positive mode. Samples collected at home for feasibility and assessed as incorrect (over- or underfilling) were excluded from the analysis. Plasma samples were measured weekly, whereas VAMS samples were analyzed monthly within the stability periods of both matrices. Stability during transportation through the postal system has also been assessed previously.^13^ Hematocrit was quantified as part of routine care at the Clinical Chemistry Laboratory of the Netherlands Cancer Institute—Antoni van Leeuwenhoek Hospital.

### Statistical Analysis

R (version 4.1.2) was used for the statistical analysis with the following additional packages: readxl, tidyverse, dplyr, ggplot2, and mcr.^21^ In case of value below the lower limit of quantitation (<LLOQ), it was assessed whether the peak was detectable with a signal-to-noise ratio (S/N) >5 for all compounds except D4A where a value >3 was used.^13^ If this ratio was less than 5 or 3, half of the LLOQ was used for analysis. If both measurements in a pair were <LLOQ with an S/N ratio lower than 5 or 3, the sample pair was excluded from the analysis. A sample pair consisted of a VAMS tube and a VAMS finger prick or a VAMS finger prick and a plasma sample.

#### Comparison VAMS Tube and VAMS Finger Prick

To determine whether there was a relative difference in the concentration between venous blood (VAMS tube) and capillary blood (VAMS finger prick), the percentage difference between the VAMS tube and VAMS finger prick was calculated (Eq. 1).

(1) $$\text{Relative}\;\text{difference}=\frac{\left(\text{VAM}S_{\text{finger prick}}-\text{VAM}S_{\text{tube}}\right)}{\left(\text{VAM}S_{\text{finger prick}}+\text{VAM}S_{\text{tube}}\right)/2}*100\%$$

Bland–Altman plots were made to illustrate the percentage difference between VAMS tube and VAMS finger prick with ±20% as upper and lower limits. When both matrices were within 20% of each other in 67% of the VAMS samples, no capillary-venous differences were considered. Although 67% within the 20% criterion was not a criterion for capillary-venous differences, we used it to determine whether the matrices could be used interchangeably.^22^

#### Conversion Methods

Two methods were used to determine the conversion of whole blood into plasma for each analyte.^12^ In the first method, the plasma concentration was predicted using Passing–Bablok (PB) regression, as this method is less sensitive to outliers.^23^ The linear regression model was predicted for each analyte and used to calculate the estimated plasma concentration (EPC_PB_) (Eq. 2). In the second method, the median conversion factor (CF) is calculated (Eq. 3). These CFs were used to calculate the EPC (EPC_CF_) (Eq. 4). Moreover, the CF was tested for hematocrit dependence using linear regression.

PB method

(2) $$\text{EP}C_{\text{PB}}=\text{intercept}+\text{slope}*C_{\text{VAMS}}$$

CF method

(3) $$\text{CF}=\text{median}\left[\frac{C_{\text{plasma}}}{C_{\text{VAMS}}}\right]$$

(4) $$\text{EP}C_{\text{CF}}=C_{\text{VAMS}}*\text{CF}$$

To compare these conversion methods, the median percentage predictive error (MPPE) and median absolute percentage predictive error (MAPE) were calculated for each method (Eqs. 5 and 6). Based on the MAPE and MPPE alongside the number of measurements in which the EPC was within ±20% of the measured plasma concentration, the best conversion method was selected for each analyte.

(5) $$\text{MPPE}=\text{median}\left[\frac{\text{EPC}-C_{\text{plasma}}}{C_{\text{plasma}}}\%\right]$$

(6) $$\text{MAPE}=\text{median}\left[\frac{\left|\text{EPC}-C_{\text{plasma}}\right|}{C_{\text{plasma}}}\%\right]$$

After identifying the best conversion method, Bland–Altman plots were constructed to illustrate the relationship between the EPC of the best conversion method and the measured plasma concentration. The difference between the EPC and plasma concentration (C_plasma_) was calculated (Eq. 7). To accept the conversion method, the difference should be within ±20% of each other.

(7) $$\text{Difference}\;\text{EPC}\;\text{en}\;C_{\text{plasma}}=\frac{\left(\text{EPC}-C_{\text{plasma}}\right)}{\left(\text{EPC}+C_{\text{plasma}}\right)/2}*100\%$$

### Feasibility

After the collection of capillary blood samples, the patients were asked if they were willing to try home sampling. Patients who participated in this optional part of the study received home sampling kits. This kit consisted of 2 VAMS devices in a clamshell in a specimen bag with a desiccant and plastic zip bag, 2 band aids, 2 alcohol wipes, 1 gauze, 2 lancets, and an instruction manual. Patients were instructed to perform the finger prick just before drug intake (ie, trough concentration) and to record both the date and time of the last drug administration and finger prick in the manual, which provided further instructions regarding sampling, packaging, and shipment. The samples were returned by regular mail in a medical envelope.

Once the sampling kit was returned to the hospital, the following items were evaluated: correct sampling time, correct use of the device, and packaging. The sampling time was considered correct if the samples were collected at the trough level. This was specified as 10–14 hours or 22–26 hours after the last dose for the dose regimen, twice a day or once a day, respectively. A complete manual is used to determine the correct sampling time. Correct use of the sampling device was verified via visual inspection. Oversampled or undersampled VAMS samples were excluded from the analyses. One device was analyzed, and the other was considered a backup sample. To ensure correct packaging, the devices had to be returned to the clamshell in the specimen bag, including the desiccant in the plastic zip bag. After the evaluation, a questionnaire was sent via e-mail. The questionnaire included the following questions: (1) Who performed the finger prick? (2) Did you manage to fill both devices? The following number of statements had to be rated from 1-fully disagree to 5-fully agree: (3A) I found the description clear. (3B) I found it easy to take finger pricks. (3C) I found it stressful to take a finger prick. (3D) I would like this to be performed more often in the future. In addition, there was the possibility of noting other comments. No formal criteria for feasibility were defined; however, the feasibility was evaluated based on the above aspects (correct use of the device, correct sampling time and packaging, and patient perspective).

## RESULTS

### Study Population and Sample Collection

A total of 153 patients were enrolled in this study. For 3 patients, no VAMS tubes were drawn because of logistic problems in the hospital. For D4A, for 1 patient, both VAMS samples were below the LLOQ with an S/N ratio of <3 and were, therefore, excluded from the tube versus finger prick analysis. Four D4A VAMS finger prick samples were below the LLOQ of 0.5 ng/mL with an S/N ratio >3 and were included in the analysis. In addition, for 1 patient, both the VAMS tube and finger prick samples were below the LLOQ with an S/N ratio >5 for sunitinib, and similar for another patient, but for N-desethyl sunitinib, allowing the data to be used.

For conversion analysis, 1 abiraterone-treated patient (incl. D4A) was excluded because both the plasma sample and VAMS finger prick were below the LLOQ, resulting in 152 patients (Table 1). For another abiraterone patient, the D4A concentrations in the plasma and VAMS samples were both below the LLOQ, but in plasma with an S/N ratio >3 and for VAMS samples below 3, it was set to 0.250 ng/mL. Similarly, for 1 sunitinib-treated patient, the plasma concentration was set to 2.5 ng/mL due to the S/N ratio was <5. In addition, for another sunitinib patient, the metabolite concentration was set to 2.5 ng/mL, due to an S/N ratio below 5. The baseline characteristics of the study population used for the conversion analysis are shown in Table 1 along with the mean concentrations of the 3 different samples.

**TABLE 1.** Baseline Characteristics, Mean Plasma Concentrations, and Mean Concentrations of VAMS Obtained After a Venipuncture and a Finger Prick

Drug	N_tot_	Age, yr [Median (Range)]	Sex, Male (%)	Hematocrit [L/L, Median (Range)]	Mean Plasma Conc. [ng/mL, (Range)]	N_VAMS_	Mean VAMS Venipuncture [ng/mL, (Range)]	Mean VAMS Finger Prick Conc. [ng/mL, (Range)]	Within ±20% (%)
Abiraterone	32	76 (55–86)	100	0.41 (0.31–0.44)	84.6 (8.43–289)	30	74.6 (6.15–288)	69.4 (6.35–272)	73
D4A	32	—	—	—	3.15 (0.46–12.8)	29	2.67 (0.477–11.4)	2.64 (0.220–12.6)	66
Alectinib	25	61 (37–82)	40	0.38 (0.29–0.50)	574 (143–1170)	25	934 (266–1750)	984 (258–1760)	92
Alectinib-M4	25	—	—	—	263 (80.0–409)	25	485 (151–855)	509 (149–829)	96
Cabozantinib	20	66 (49–81)	80	0.42 (0.32–0.56)	717 (250–1350)	20	496 (236–908)	516 (240–1120)	90
Imatinib	36	67 (47–83)	53	0.38 (0.27–0.46)	1502 (639–3210)	36	1303 (629–1990)	1262 (558–2030)	89
N-desmethyl imatinib	36	—	—	—	364 (164–683)	36	294 (153–525)	285 (143–505)	94
Olaparib	18	66 (49–83)	11	0.37 (0.30–0.44)	3903 (827–6810)	17	2965 (531–5740)	2905 (506–5560)	94
Sunitinib	22	65 (47–84)	50	0.41 (0.34–0.51)	31.7 (2.50–81.6)	22	61.8 (2.15–173)	62.7 (2.90–170)	95
N-desethyl sunitinib	22	—	—	—	18.9 (2.50–39.3)	22	25.0 (3.07–46.0)	26.4 (3.65–54.5)	100

N_tot_, number of included patients; N_VAMS_, number of pairs used for comparison between VAMS venipuncture and finger prick.

### Comparison VAMS Tube and VAMS Finger Prick

In total, 150 sample pairs were included in the comparison between the VAMS tube and VAMS finger prick, of which 112 also included active metabolites. The differences between the analyte concentrations measured in the VAMS tube and the VAMS finger prick were calculated using equation 1: The International Council for Harmonisation (ICH) M10 guidelines for cross-validation were met for all analytes except for D4A. The results are shown in Table 1, and the Bland–Altman plots are shown in **Supplemental Digital Content 1** (see **Figure**, http://links.lww.com/TDM/A826). Ninteen out of 29 pairs for D4A were within ±20%, equivalent to 66%. Four data points of D4A had 1 or both <LLOQ measurements, as highlighted in **Supplemental Digital Content 1** (see **Figure**, http://links.lww.com/TDM/A826).

### Conversion Methods

#### PB Regression and CFs

**FIGURE 1.**
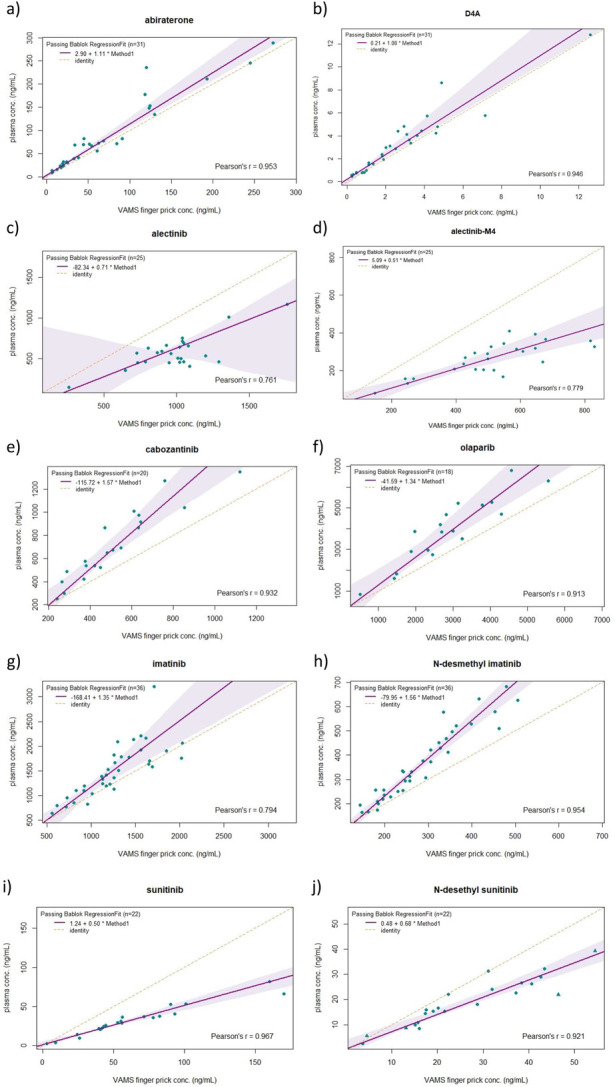
PB regression for abiraterone (A), D4A (B), alectinib (C), alectinib-M4 (D), cabozantinib (E), olaparib (F), imatinib (G), N-desmethyl imatinib (H), sunitinib (I), and N-desethyl sunitinib (J). Dotted line = identity; solid line = regression. Triangles in J indicate that the quality controls in the analytical run did not meet the requirements.

The CF and PB regression were calculated and are listed in Table 2. Figure 1 shows the PB regression curves for all agents. Correlation coefficient was very high (ie, 0.90–1.00) for abiraterone, D4A, cabozantinib, N-desmethyl imatinib, olaparib, and N-desethyl sunitinib, and high (ie, 0.70–0.90) for alectinib, alectinib-M4, and imatinib. The CF was below one for alectinib, alectinib-M4, sunitinib, and N-desethyl sunitinib, indicating a greater affinity for the red blood cell compartment. Other drugs and metabolites showed greater affinity for the plasma compartment, resulting in a CF above one (Table 2).

**TABLE 2.** Conversion Results for VAMS to Plasma for PB and CF

Drug	N	Passing–Bablok Regression				CF	
		Slope (95% CI)	Intercept (95% CI)	Correlation Coefficient	EPC Within ±20% [N (%)]	CF (95% CI)	EPC Within ±20% [N (%)]
Abiraterone	31	1.11 (1.02 to 1.28)	2.90 (−0.76 to 6.83)	0.953	20 (65)	1.23 (0.90 to 2.05)	20 (65)
D4A	31	1.08 (0.99 to 1.40)	0.21 (−0.23 to 0.36)	0.946	20 (65)	1.22 (0.89 to 2.21)	17 (55)
Alectinib	25	0.71 (−0.41 – 1.06)	−82.3 (−457 to 931)	0.761	14 (56)	0.60 (0.37 to 0.78)	15 (60)
Alectinib-M4	25	0.51 (0.36 to 0.70)	5.09 (−59.1 to 67.2)	0.779	17 (68)	0.53 (0.35 to 0.68)	18 (72)
Cabozantinib	20	1.57 (1.14 to 1.88)	−116 (−246 to 106)	0.932	16 (80)	1.36 (1.06 to 1.77)	15 (75)
Imatinib	36	1.35 (1.00 to 1.84)	−168 (−705 to 150)	0.794	28 (78)	1.19 (0.87 to 1.64)	30 (83)
N-desmethyl imatinib	36	1.56 (1.37 to 1.74)	−80.0 (−124 to −35.6)	0.954	31 (86)	1.25 (1.01 to 1.55)	34 (94)
Olaparib	18	1.34 (1.03 to 1.72)	−41.6 (−811 to 1044)	0.913	14 (78)	1.33 (1.09 to 1.83)	13 (72)
Sunitinib	22	0.50 (0.41 to 0.58)	1.24 (−1.67 to 5.32)	0.967	18 (82)	0.53 (0.38 to 0.74)	18 (82)
N-desethyl sunitinib	22	0.68 (0.57 to 0.80)	0.48 (−1.72 to 3.86)	0.921	16 (72)	0.70 (0.50 to 1.10)	16 (72)

CI, confidence interval; EPC, estimated plasma concentration.

#### Evaluation of Conversion Methods

The evaluation results for both conversion methods are shown in Table 2 (EPC) and **Supplemental Digital Content 1** (see **Table**, http://links.lww.com/TDM/A826) (MAPE and MPPE). For most of the analytes, no clear differences were observed between the 2 conversion methods. The CF method was slightly better for imatinib and its metabolite, whereas for D4A, the PB regression seemed more suitable (Table 2 and see **Table**, **Supplemental Digital Content 1**, http://links.lww.com/TDM/A826). Although the MAPEs of abiraterone, D4A, and alectinib were within 20% (see **Table**, **Supplemental Digital Content 1**, http://links.lww.com/TDM/A826), the differences between the measured plasma concentrations and EPC were not within ±20% for either conversion method (Table 2).

**FIGURE 2.**
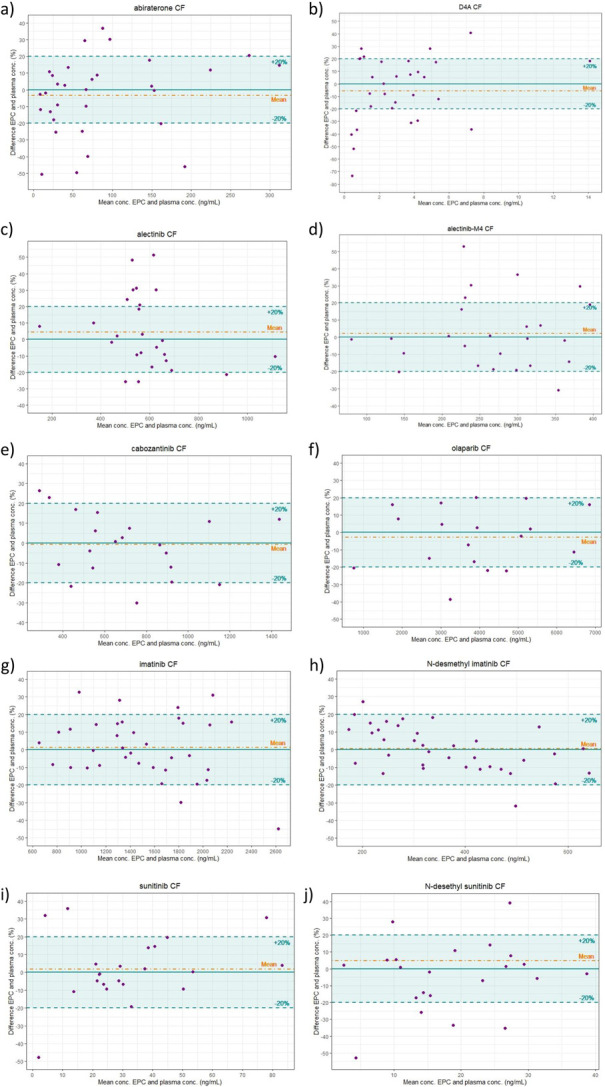
Bland–Altman plot comparing estimated plasma concentrations with measured plasma concentrations for abiraterone (A), D4A (B), alectinib (C), alectinib-M4 (D), cabozantinib (E), olaparib (F), imatinib (G), N-desmethyl imatinib (H), sunitinib (I), and N-desethyl sunitinib (J). Solid line = zero-difference; dot-dashed line = mean difference; dashed lines = ±20% difference.

Bland–Altman plots were constructed for all drugs using the CF method (Fig. 2). The established conversion methods fulfilled the ICH M10 criteria for alectinib-M4, cabozantinib, imatinib, N-desmethyl imatinib, olaparib, sunitinib, and N-desethyl sunitinib. Neither conversion method met the ICH M10 criteria for abiraterone, D4A, or alectinib when comparing EPCs to plasma concentrations.

The CF was found to be significantly dependent on the hematocrits of cabozantinib, imatinib, and N-desmethyl imatinib. The minimum and maximum calculated CFs, compared with the estimated CFs in Table 2, based on the minimum and maximum hematocrit values in each particular population were −16% and 28% for cabozantinib, −21% and 17% for imatinib, and −12% and 11% for N-desmethyl imatinib.

### Feasibility Home Sampling

Of 153 patients included in the study, 119 (78%) were willing to undergo home sampling. Of these, 94 (61%) collected samples at home (Table 3). Of the 25 patients for whom samples were not collected, 3 switched therapy, 5 did not receive the sampling kit because of logistic problems, and 17 did not return the samples. The reasons for patients declining to try it at home included a desire to avoid dealing with the disease at home, lack of time or energy, and fear of needles.

**TABLE 3.** Results of the Feasibility by Visual Examination (N = 94) and Questionnaire (N = 74)

Drug	N home Collections (of Inclusions, %)	Both Samples Correct, n	One Sample Correct, n	No Samples Correct, n	Correct Packaging (%)	Correct Sampling Time (%)	VAMS Conc. [Mean, ng/mL (Range)]	VAMS Conc. Metabolite [Mean, ng/mL (Range)]
Abiraterone	16 (50)	12	4	0	56	56	85.0 (5.69–859)	2.85 (0.251–19.3)
Alectinib	17 (68)	13	4	0	71	71	812 (9.0–1360)	452 (28.0–805)
Cabozantinib	9 (45)	4	4	1	89	44	390 (185–753)	—
Imatinib	21 (58)	15	5	1	81	71	966 (464–1780)	207 (114–454)
Olaparib	12 (68)	8	3	1	75	78	1387 (202–4170)	—
Sunitinib	19 (86)	14	0	5	79	68	71.3 (1.91–184)	30.4 (3.93–67.5)
Total	94 (61)	66	20	8	74	64		

Questionnaire (1 = Fully Disagree, 5 = Fully Agree)	1 (%)	2 (%)	3 (%)	4 (%)	5 (%)
I found the description clear	3	3	5	24	65
I found taking the finger prick easy	1	0	7	32	59
I found it not stressful to take the finger prick	9	19	30	14	28
I would like it to be done this way more often in the future	0	0	36	31	32

Two home-sampled VAMS devices were visually examined. Patients were able to fill both devices properly in 70% of cases. In 21% of the instances, 1 sample was accepted, and 1 was underfilled or overfilled and rejected. Both samples were rejected in 9% of cases. Notably, 99% of patients answered that they managed to fill both devices properly. The samples were sent in correct packaging in 74% of the cases. Minor packaging errors included an open aluminum bag or the absence of a plastic zip bag. Samples were collected at the correct sampling time (trough level) in 64% of the patients. The rejected home-sampled VAMS devices were not analyzed. In 2 abiraterone home samples, neither abiraterone nor D4A were detectable, whereas in 1 abiraterone home sample, only D4A was not detectable. However, home samples in which the analyte was detectable, despite being below the LLOQ, are displayed. These included 1 sample for D4A, 1 for alectinib and its metabolite, 1 for olaparib, and 2 for sunitinib.

Of the 94 patients who underwent home sampling, 75 (79%) completed the questionnaire. Most patients performed the finger prick themselves (76%), and the majority performed this task at the correct sampling time (69%). Patients were positive toward the instruction manual (89% agree or fully agree) and thought it was easy to take the finger prick (92% agree or fully agree), and neutral to positive toward using home sampling more often in the future (64% fully agree or agree, 36% neutral). Patients had varying perceptions of the stressfulness of performing a finger prick, with scores ranging from 1 to 5 in an evenly distributed manner. See Table 3 for a summary of the questionnaire and visual examination and the measured concentrations in the home-sampled VAMS devices.

## DISCUSSION

In this study, the methods for converting VAMS into plasma were established. These criteria met the acceptance criteria for alectinib-M4, cabozantinib, imatinib, N-desmethyl imatinib, olaparib, sunitinib, and N-desethyl sunitinib. These conversion methods were established by collecting samples in a clinical validation setting and are, therefore, applicable to clinical practice after further validation in an independent cohort. The feasibility of home sampling with VAMS by cancer patients for the application of TDM or oral targeted anticancer drugs was demonstrated. Furthermore, there is no clinically relevant difference between venous and capillary whole blood for all drugs and metabolites.

No conversion method could be validated according to the guidelines for abiraterone, D4A, and alectinib: the estimated plasma concentrations were not within ±20% for at least 2/3rd of the patients. Specifically for abiraterone, it is regrettable that the conversion results did not meet the requirements, given its instability in wet plasma and the need for transportation on dry ice. The use of VAMS in its dry form is an elegant solution to this problem. Abiraterone has a high peak-to-trough ratio. The trough levels were typically in the lower range (2–40 ng/mL), whereas in this clinical validation, samples were collected across the pharmacokinetic curve, including peak concentrations. To confirm the concentrations around the therapeutic target, additional trough-level samples should be collected before implementing this method in clinical practice.

Alectinib conversion did not meet the criteria, as only 60% of EPCs and actual plasma measurements were within ±20% of each other; therefore, the validation was unsuccessful. Notably, alectinib-M4 conversion met the criteria despite both compounds having similar characteristics. For example, the FDA review reported the average blood-to-plasma concentration ratio to be 1.3–2.9 for alectinib and 2.5–2.6 for M4.^24^ The wide 95% confidence interval in the lower and higher alectinib ranges was likely due to the limited concentration range observed, with only 2 VAMS concentrations below 500 ng/mL and above 1500 ng/mL.

According to the International Association of Therapeutic Drug Monitoring and Clinical Toxicology guidelines for dried blood–based sampling methods, a minimum of 40 patients is needed for a coefficient of variation >5% and a range ratio >25 for clinical validation studies.^12^ Ideally, this should be followed by testing using an external validation cohort. Unfortunately, it is not feasible to collect this number of patients in clinical practice for oral targeted anticancer agents. Sample size could potentially play a role in meeting these guidelines. Applying the established conversion method to the same dataset from which it was derived represents an ideal scenario but does not fully address the robustness of the method. However, we did not succeed in establishing a method for abiraterone or alectinib treatment. The jackknife method is another option when no independent validation cohort is available, and a sample size of 40 is not reached.^12^ However, for alectinib, the concentration range was too limited to draw definitive conclusions, whereas for abiraterone, the number of samples around the clinical cutoff value was relatively low. Therefore, further validation using independent datasets is necessary to substantiate the applicability of our conversion method in clinical practice.

Figures 1 and 2 show the PB regression curves and Bland–Altman plots, respectively, for the CF method. Although a good correlation was observed in the PB regression curves for all analytes, the Bland–Altman plots provided clear insights into the relative deviations. For example, in the case of abiraterone, the correlation in the low concentration range seems very good, whereas the Bland–Altman plot shows that these deviations increase to 50%. On the other hand, for imatinib, the correlation in the PB regression curve seemed slightly worse than that for abiraterone, whereas in the Bland–Altman regression curve, the maximum deviation was approximately 30% (Figs. 1 and 2). The 2 different plots complement each other well, providing valuable insights into the reliability of the established method.

Although hematocrit values were found to be significant in the linear regression analyses for cabozantinib, imatinib, and its metabolite, they were omitted from the conversion process because of the impracticality of requiring hematocrit values, particularly in the context of home sampling. Despite its significance, the inclusion of hematocrit was not shown to be essential in the conversion of VAMS to plasma to meet the criteria. Notably, these 3 analytes did not show preference for the red blood cell compartment. Analytes with a higher affinity for the red blood cell compartment, such as alectinib and sunitinib, are expected to show greater dependency on the hematocrit.

In an earlier study by Krützmann et al, VAMS was applied for the TDM of imatinib, and N-desmethyl imatinib, a CF of 1.28 was used, in a DBS clinical validation study.^10,14^ The CF of 1.19 found in our study is in line with earlier applications of blood-to-plasma conversion. Mukai et al discovered a regression formula remarkably similar to ours, although they utilized weighted Deming regression. Their formula yielded a slope and intercept of 1.36 and −164, respectively, compared with our values of 1.35 and −168.^9^ Although this multiplier and regression formula were found in DBS but not in VAMS samples, it is the same concept of converting whole blood to plasma concentration. Verougstraete et al^25^ calculated the difference between plasma and whole blood concentrations of different hematological kinase inhibitors, including imatinib, without the use of a microsampling device. This involved a hematocrit-corrected ratio of 1.15, which was comparable with our blood-to-plasma ratio. Zimmermann et al^7^ investigated the blood-to-plasma conversion of different kinase inhibitors using VAMS including cabozantinib, using VAMS and found a factor of 1.852 in their clinical validation study, which differs substantially from the factor of 1.36 found in our study. In an earlier in vitro study conducted by Zimmermann et al,^6^ a VAMS/plasma ratio of 0.72 was identified, corresponding to a CF of 1.39, which is in agreement with our findings. The concentration ranges, ages, and hematocrit values were similar between the 2 clinical validation studies. Although 2 patients in our study had hematocrit values of 0.52 and 0.56 L/L, compared with a maximum level of 0.47 L/L in their study. This still does not comply with each other, as 1.852 is not in our 95%CI of the CF of cabozantinib: 1.36 (1.06–1.77), so even at these high hematocrit levels we did not estimate such a high factor. In their report on the analytical and clinical validation of olaparib in DBS, Canil et al^8^ determined that DBS concentrations should be divided by 0.78, which corresponds to a CF of 1.28 from DBS to plasma. This is consistent with the VAMS CF of 1.33.

For all analytes except D4A, there was no difference between the VAMS samples collected from the tube and those collected after the finger prick. Capillary blood is the equilibrium between venous and arterial blood. In general, differences between capillaries and venously drawn blood are expected for drugs with short half-lives and those sampled during the absorption phase.^11^ However, this was not the case for our selected drugs, and the goal of using a home-sampling device to obtain trough levels. However, testing was valuable because the calibration standards and quality controls were made with EDTA whole blood, and patient samples were not. For D4A, the deviation between VAMS tube and finger seemed more present in the lower concentration range, where the absolute difference is not considered clinically relevant, although relative difference is greater than ±20%. The lack of a stable isotopically labeled internal standard for D4A in the bioanalytical method may have contributed to this.^13^

The results of the feasibility evaluation indicated that a considerable number of people were interested in using home sampling. The design was intentionally noncommittal, and people were asked if they wanted to try it at home only after finger pricking in the hospital. The patients did not receive training but received only the instruction manual. Ultimately, 94/153 people participated in the study. At least 1 sample was analyzed in 91% of the cases. Instructions on collection time were initially given verbally but were added to the manual halfway through the study, after which the timing of collection improved. It would be beneficial to provide further clarification of the instructions for packaging and shipping the sampling kit, as this was not fully accurate in 26% of the cases. The participants were patients with cancer that was spread over a wide range of ages. The patients also knew that the VAMS samples were drawn for research purposes and that the results would not be reported to the treating physician, in contrast to the routine TDM service. A small selection bias may have been present because we first asked the doctors which patients could be contacted. However, the participating patients were representative of patients who may use this in the future.

The feasibility of VAMS home sampling in an oncological setting was also tested by both earlier mentioned groups of Zimmermann et al and Krützmann et al.^7,14^ The various groups employed different approaches, with varying levels of instruction and training. Although our patients received only the instruction manual after the procedure in the hospital, patients in Brazil also received video instruction, and in Germany, patients were trained and required to take the first sample under supervision, and the instruction manual. In a study by Zimmermann in Germany, the acceptance ratio was 93.1%, whereas in our case, it was 81%, and in Krützmann in Brazil, it was 78%. The ratios were calculated by dividing the number of accepted samples by the total number of samples collected. However, when only 1 sample was needed for analysis, the ratios for Brazil and our study were 94% and 91%, respectively, which are considered sufficient and similar to those for Germany. Notably, patients in our study were required to collect 2 samples from 1 finger prick, whereas in Germany, 4 samples were collected: one in the hospital and 3 at different time points at home. Collecting 2 samples in a single row can be challenging in cases of low blood flow.

The next step will be the external validation of the conversion methods, followed by the implementation of home sampling with VAMS for TDM. This will be available to those who wish to use it instead of venipuncture in outpatient clinics. This approach offers the possibility of interim monitoring without the need for a hospital visit, facilitates earlier dose adjustments, and provides timely results for discussion during consultation with a medical oncologist. In addition, it increases the likelihood of obtaining a reliable TDM sample by allowing patients to obtain trough levels at home without adjusting their medication intake timing. This eliminates the inconvenience of scheduling outpatient appointments and blood draws, which may coincide with the drug absorption phase because blood is collected immediately after drug intake.

## CONCLUSION

This study established VAMS conversion methods for alectinib-M4, cabozantinib, imatinib, N-desmethyl imatinib, olaparib, sunitinib, and N-desethyl sunitinib that met the acceptance criteria, which should be validated in an independent cohort. Further research is required to apply VAMS to abiraterone, D4A, and alectinib. The feasibility of home sampling of patients using these anticancer agents was demonstrated. The next step involves validating the established conversion methods with an independent cohort, followed by integrating home sampling via VAMS for TDM, thereby providing patients with an alternative to venipuncture at the outpatient clinic.

## Supplementary Material

SUPPLEMENTARY MATERIAL
