# Supplementary material for: Clinical Application of Volumetric Absorptive Microsampling for Therapeutic Drug Monitoring of Oral Targeted Anticancer Drugs
Source: Ther Drug Monit. 2025 Feb 25;47(5):625–34. doi: 10.1097/FTD.0000000000001315 (PMC12422603; doi:10.1097/FTD.0000000000001315)
Supplement: SUPPLEMENTARY MATERIAL [file tdm-47-625-s001.pdf]

Clinical application of Volumetric Absorptive Microsampling for Therapeutic Drug Monitoring of oral targeted anticancer drugs

M. Meertens[1], N. Kerssemakers[1], N. de Vries[1], H. Rosing[1], N. Steeghs[2,3], J.H. Beijnen[1,4], A.D.R. Huitema[1,5,6]

[1]Department of Pharmacy & Pharmacology, The Netherlands Cancer Institute – Antoni van Leeuwenhoek Hospital, Amsterdam, The Netherlands.

[2]Department of Medical Oncology, The Netherlands Cancer Institute - Antoni van Leeuwenhoek Hospital, Amsterdam, The Netherlands

[3]Department of Medical Oncology, Utrecht University Medical Centre, Utrecht, The Netherlands

[4]Division of Pharmacoepidemiology and Clinical Pharmacology, Utrecht Institute for Pharmaceutical Sciences, Utrecht University, Utrecht, The Netherlands

[5]Department of Clinical Pharmacy, University Medical Center Utrecht, Utrecht University, Utrecht, The Netherlands

[6]Department of Pharmacology, Princess Máxima Center for Pediatric Oncology, Utrecht, The Netherlands

Correspondence: Alwin D.R. Huitema; Department of Clinical Pharmacy, University Medical Center Utrecht, Utrecht, The Netherlands (e-mail: ahuitem8@umcutrecht.nl)

Supplemental Material

Supplemental Table 1*: Predictive performance of two conversion methods. MAPE: median absolute predictive error; MPPE: median percentage predictive error; CI = confidence interval.*

		Passing-Bablok				Conversion factor			
drug	N	MPPE (%)	95%CI	MAPE (%)	95%CI	MPPE (%)	95%CI	MAPE (%)	95%CI
**abiraterone**	31	0.23	(-41.6 - 28.9)	11.1	(0.23 - 41.6)	0.28	(-39.8 - 38.1)	12.5	(0.31 - 41.6)
**D4A**	31	-0.08	(-31.9 - 39.0)	14.0	(1.00 - 39.0)	0.12	(-44.3 - 37.6)	19.5	(4.13 - 51.9)
**alectinib**	25	0.14	(-25.7 - 76.5)	12.5	(0.19 - 76.5)	-0.77	(-22.7 - 65.7)	15.4	(1.35 - 65.7)
**alectinib-M4**	25	-0.92	(-22.8 - 52.2)	15.8	(0.70 - 52.2)	-0.77	(-21.7 - 55.6)	15.3	(0.71 - 55.6)
**cabozantinib**	20	-0.12	(-29.6 - 19.8)	10.0	(1.21 - 29.6)	0.07	(-23.1 - 28.4)	11.7	(0.88 - 28.5)
**imatinib**	36	0.17	(-25.2 - 38.2)	12.9	(0.90 - 28.7)	0.37	(-27.3 - 36.9)	10.8	(1.01 - 36.9)
**N-desmethyl imatinib**	36	-0.10	(-23.6 - 24.0)	7.21	(0.67 - 25.9)	-0.19	(-18.8 - 23.2)	10.0	(1.01 - 27.9)
**olaparib**	18	0.03	(-28.7 - 22.0)	15.8	(2.10 - 28.7)	0.08	(-27.1 - 22.1)	16.4	(2.06 - 28.1)
**sunitinib**	22	-0.69	(-15.1 - 56.9)	7.19	(0.37 - 56.9)	-0.22	(-27.5 - 40.8)	7.77	(0.66 - 41.0)
**N-desethyl sunitinib**	22	-0.58	(-32.5 - 40.2)	11.8	(0.96 - 40.3)	-0.50	(-35.6 - 40.2)	9.84	(1.15 - 45.0)

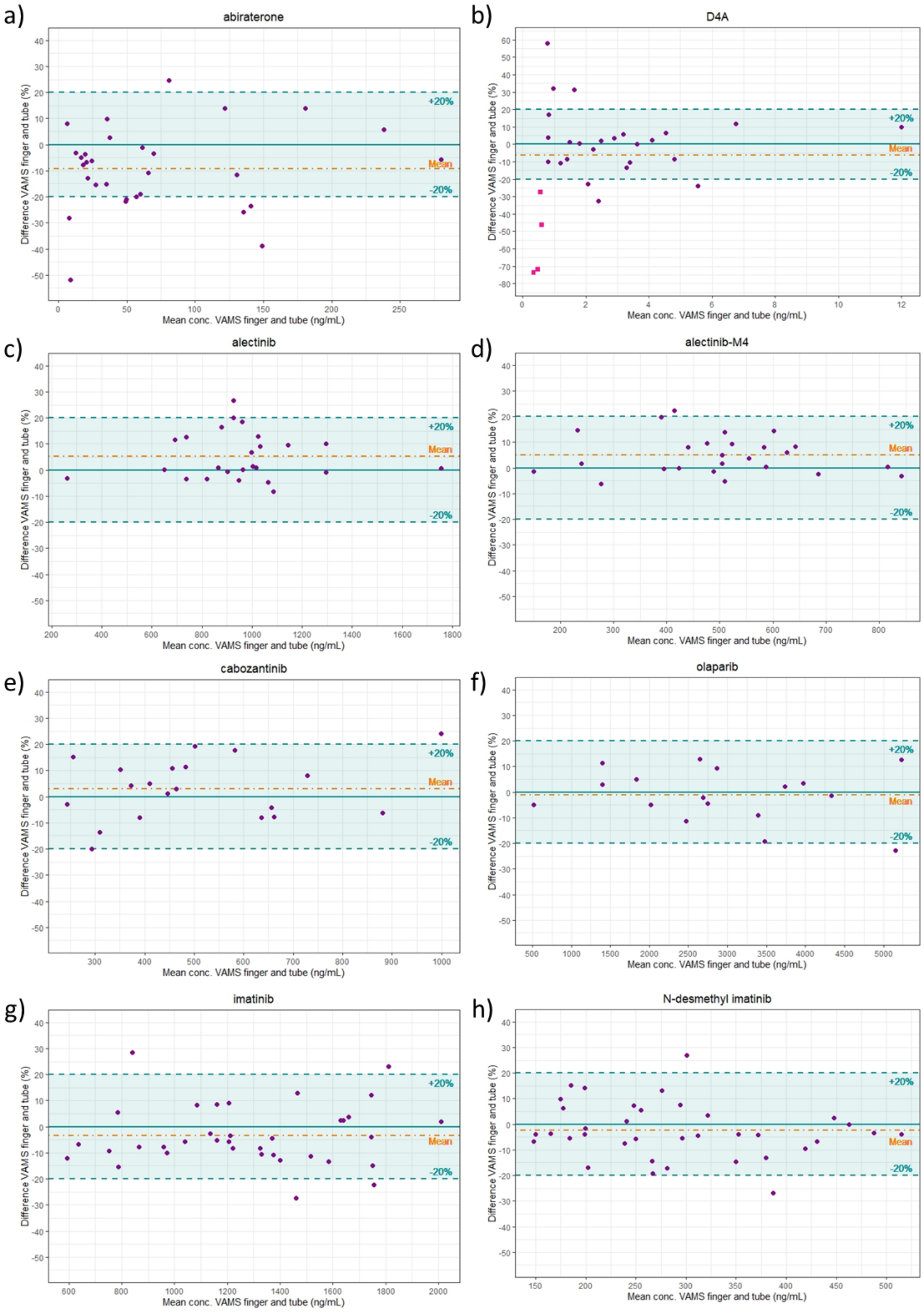

a)
abiraterone
b)
D4A
c)
alectinib
d)
alectinib-M4
e)
cabozantinib
f)
olaparib
g)
imatinib
h)
N-desmethyl imatinib
Difference VAMS finger and tube (%)
Mean conc. VAMS finger and tube (ng/mL)
+20%
Mean
-20%

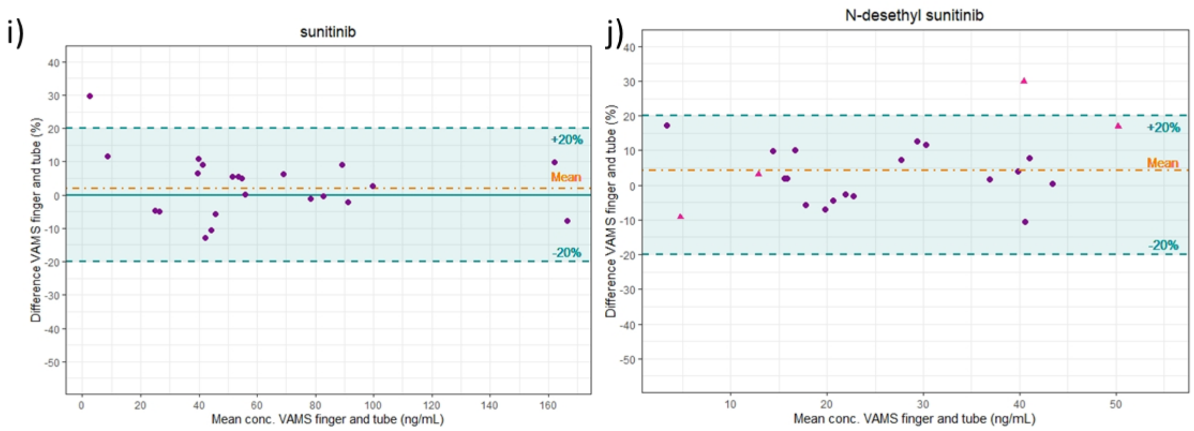

Supplemental Figure 1: *Difference between VAMS drawn from the finger and tube, presented in Bland-Altman plots for abiraterone (a), D4A (b), alectinib (c), alectinib-M4 (d), cabozantinib (e), olaparib (f), imatinib (g), N-desmethyl imatinib (h), sunitinib (i) and N-desethyl sunitinib (j). Solid lline = zero; dot-dashed line: mean difference; dashed lines = ±20% difference. Square = one or both measurements below LLOQ (0.5 ng/mL), triangle = quality controls did not meet requirements in analytical run.*
